# Suppressive myeloid cells in SARS-CoV-2 and *Mycobacterium tuberculosis* co-infection

**DOI:** 10.3389/fimmu.2023.1222911

**Published:** 2023-07-20

**Authors:** Jane Alexandra Shaw, Stephanus T. Malherbe, Gerhard Walzl, Nelita du Plessis

**Affiliations:** Department of Science and Technology/National Research Foundation (DSI-NRF) Centre of Excellence for Biomedical Tuberculosis Research, South African Medical Research Council Centre for Tuberculosis Research, Biomedical Research Institute, Division of Molecular Biology and Human Genetics, Faculty of Medicine and Health Sciences, Stellenbosch University, Cape Town, South Africa

**Keywords:** myeloid derived suppressor cells, tuberculosis, SARS-CoV-2, COVID-19, coinfection

## Abstract

Epidemiologic data show that both current and previous tuberculosis (TB) increase the risk of in-hospital mortality from coronavirus disease-2019 (COVID-19), and there is a similar trend for poor outcomes from *Mycobacterium tuberculosis* (Mtb) infection after recent SARS-CoV-2. A shared dysregulation of immunity explains the dual risk posed by co-infection, but the specific mechanisms are being explored. While initial attention focused on T cell immunity, more comprehensive analyses revealed a dysfunctional innate immune response in COVID-19, characterized by reduced numbers of dendritic cells, NK cells and a redistribution of mononuclear phagocytes towards intermediate myeloid subsets. During hyper- or chronic inflammatory processes, activation signals from molecules such as growth factors and alarmins lead to the expansion of an immature population of myeloid cells called myeloid-deprived suppressor cells (MDSC). These cells enter a state of pathological activation, lose their ability to rapidly clear pathogens, and instead become broadly immunosuppressive. MDSC are enriched in the peripheral blood of patients with severe COVID-19; associated with mortality; and with higher levels of inflammatory cytokines. In TB, MDSC have been implicated in loss of control of Mtb in the granuloma and ineffective innate and T cell immunity to the pathogen. Considering that innate immune sensing serves as first line of both anti-bacterial and anti-viral defence mechanisms, we propose MDSC as a crucial mechanism for the adverse clinical trajectories of TB-COVID-19 coinfection.

## 1 Introduction

There is now ample evidence that regions with a high prevalence of tuberculosis (TB) disease and latent TB infection (LTBI, where an asymptomatic person has a positive interferon-γ release assay or skin test), also have a high prevalence of recent SARS-CoV-2 infection (1–4). As a result, acute or chronic coinfection, or acute sequential infection with *Mycobacterium tuberculosis* (Mtb) and SARS-CoV-2 has become inevitable.

We know from epidemiologic reports during the pandemic that coinfection with Mtb and SARS-CoV-2 worsens patient outcomes. Both current active TB disease (defined as culture, molecular test, or other Mtb test positive, with symptoms or imaging changes that justify the initiation of full TB treatment) and previous TB, increase the risk of in-hospital mortality from coronavirus disease-2019 (COVID-19), and the case fatality rate for coinfection is higher than for COVID-19 alone (5, 6). The lymphopaenia which characterizes COVID-19 is exaggerated in coinfection, and markers of inflammation such as D-dimer and ferritin are increased over and above the COVID-19 levels (7, 8). Transcriptomics and RNAseq data from whole blood, peripheral blood mononuclear cells (PBMC) and bronchoalveolar lavage fluid (BALF) of patients with COVID-19, and those with TB across the clinical spectrum, has shown that there is similarity in the immunopathogenesis of the two diseases through commonly enriched genes in 12-gene disease-exacerbation hot spots (9). Moreover, the inflammatory milieu from Mtb-infected human macrophages increased SARS-CoV-2 infection *in vitro*, which correlated with IFNA1, IFNB1, IFNG, TNF, and other inflammatory gene induction. Interestingly, in Mtb-infected mice, superinfection with SARS-CoV-2 resulted in increased Mtb dissemination, but a lower SARS-CoV-2 viral load in the tissues (10). In a different murine study, the protective effect of pre-existing Mtb infection on the pathological consequences of SARS-CoV-2 occurred without adversely affecting TB outcomes (11). This collection of findings shows that Mtb infection increases the risk of severe COVID-19 in humans, and suggests the possibility that SARS-CoV-2 coinfection may also trigger the progression of subclinical TB to TB disease.

Patients with active TB disease and SARS-CoV-2 coinfection have lower numbers of Mtb-specific T cells (12). They also produce less IFN-γ and other proinflammatory cytokines, chemokines and growth factors on SARS-CoV-2 stimulation; produce less interferon-y (IFN-y) and several other cytokines on Mtb stimulation (though to a lesser extent than the reduction on SARS-CoV-2 stimulation); and have different overall cytokine signatures compared to infection with each pathogen alone (12–14). One possible mechanism underlying this observed immune suppression in the presence of chronic stimulation by Mtb or SARS-CoV-2 antigens, is the presence of suppressive myeloid cells such as myeloid-derived suppressor cells (MDSC). MDSC are known to inhibit many immune pathways, particularly T cell responses. They have now been both directly and indirectly implicated in the pathogenesis of both COVID-19 and TB (15–17). However, their role in coinfection is yet to be explored. In this article we will introduce the reader to MDSC, briefly review the evidence for their involvement in TB disease and COVID-19, and then discuss the potential role of these cells in determining the outcome of Mtb/SARS-CoV-2 coinfection.

## 2 Suppressive myeloid cells

Suppressive myeloid cells are considered critical in immune regulation and tolerance, maintaining the delicate balance between healing and harm during the immune response. They limit excessive inflammation and prevent immune-mediated tissue damage in the early response to a tissue insult, promote immune tolerance during tissue repair or pregnancy, and augment protective anti-pathogen responses in acute infection (18–22). However, in pathological conditions such as chronic inflammation, cancer, or extensive tissue trauma, the scales tip toward more harm than help. The function of MDSC in the pathophysiology of cancer is well described (23–25), but in chronic infection and respiratory disease is still in the early stages of investigation. Our understanding of their role in these conditions is hampered by a few ongoing issues. Firstly, the cell type terminology is not globally accepted. Secondly, the nature of MDSC remains poorly defined, partly because these cells likely differentiate into suppressive macrophage subsets upon entering tissue sites. However, most agree that MDSC are cells of myeloid origin which acquire a state of pathological (or alternative) activation in response to the prolonged weak pro-inflammatory signals that are present in chronic infection or cancer (25–27). As a result, they lose their ability to rapidly and effectively clear pathogens, instead becoming immunosuppressive by inhibiting natural killer (NK) cell, B cell, and T cell responses, amongst others.

**Figure 1:**
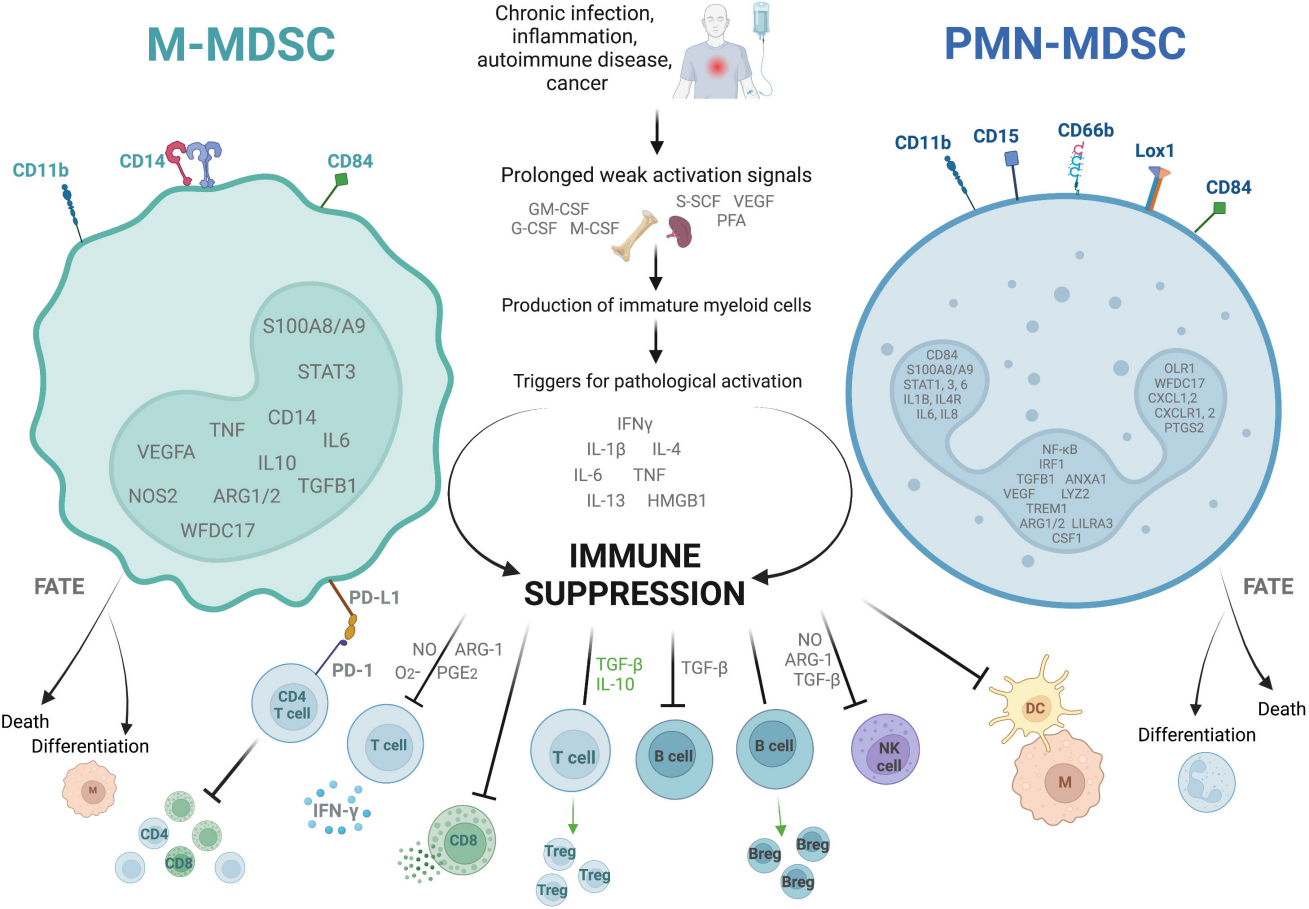
Characteristics of the two dominant subtypes of myeloid derived suppressor cells. The figure shows the two main types of myeloid derived suppressor cells (MDSC), monocytic (M)- and polymorphonuclear (PMN)-MDSC. They are identified as cells in the low density portion of a Ficoll gradient with a low expression of HLA-DR, as well as specific combinations of cell surface markers, and the upregulation of specific genes (shown inside the nucleus). They arise in situations of prolonged weak activation signals, by which they are pathologically activated to become immunosuppressive. They exert their immunosuppressive effects through several different mechanisms including suppression of CD4^+^ and CD8^+^ T cell differentiation, cytokine release, and cytotoxic degranulation; promotion of T regulatory cell (Treg) and B regulatory cell (Breg) differentiation and function; and inhibition of B cell, natural killer cell (NK), and antigen presenting cell (M, macrophage; DC, dendritic cell) functions. They are short lived, either dying after a few days in circulation or differentiating into suppressive macrophages or suppressive neutrophils. ARG-1, Arginase-1; G-CSF, granulocyte colony-stimulating factor; GM-CSF, granulocyte-macrophage colony-stimulating factor; HMGB-1, high mobility box group-1; IFNγ, interferon-γ; IL, interleukin; Lox-1, lectin- type oxidized LDL receptor 1; M-CSF, macrophage colony- stimulating factor; NO, nitric oxide; PD-1, programmed cell death protein-1; PD-L1, programmed death ligand-1; PFA, polyunsaturated fatty acids; PG-E_2,_ prostaglandin-E_2_; SCF, stem cell factor; TGF-β, transforming growth factor-β; TNF, tumor necrosis factor; VEGF, vascular endothelial growth factor. Created with BioRender.com.

MDSC are generally divided into two main subtypes (**Figure 1**). They are named for their cell lineage of origin as polymorphonuclear or granulocytic MDSC (PMN-MDSC) and monocytic MDSC (M-MDSC). A third group which comprises only a small proportion of the total MDSC population is known as ‘early MDSC’, and consists of potent immunosuppressive myeloid progenitors (28). Other subtypes such as eosinophilic MDSC have been proposed, but are not well characterized as yet. In circulation, both PMN- and M-MDSC have a short lifespan of a few days, though the latter survive longer *in vitro* (29) and the half-life may well be prolonged in inflammatory states (30). Their continuous recruitment to tissues is what results in long term effects (31). In tumors, M-MDSC rapidly differentiate into tumor-associated macrophages (TAMs) which are associated with tumor progression, and inflammatory dendritic cells (32, 33). Tumor associated neutrophils (TANs) are a heterogeneous population of cells which, in mice, includes both neutrophils with anti-tumor (N1) and suppressive/pro-tumor (N2) properties, the latter sharing some cell surface markers and biochemical properties with PMN-MDSC (34, 35).

Identifying these cells is complex, and not always consistent between studies. Cell surface markers which identify MDSC differ between mice and humans. For the purpose of this review we will focus on human-relevant markers only. PMN-MDSC are identified as CD11b^+^CD14^-^CD15^+^/CD66b^+^ cells in the low density Ficoll gradient fraction of PBMC. Other marker combinations have been proposed which do not need a Ficoll gradient, such as CD15^+^/CD66b^+^CD14^-^LOX1^+^ and CD15^+^/CD66b^+^CD14^-^CD84^+^. All human MDSC are HLA-DR^lo/-^. M-MDSC are identified as CD11b^+^CD14^+^CD15^-^HLA-DR^lo/-^ cells in the low density Ficoll fraction of PBMC, or alternatively CD14^+^/CD66b^-^CXCR1^+^ or CD14^+^/CD66b^-^CD84^+^ (28, 31, 36). Early MDSC are identified as Lin^-^HLA-DR^-^CD33^+/hi^ (where Lin is CD3, CD14, CD15, CD19 and CD56) (25, 28). Some of the newer markers such as LOX-1 have yet to be validated in infection-induced MDSC.

Classical neutrophils and monocytes are activated by pathogen- and damage-associated molecular patterns (such as lipopolysaccharide and heat shock proteins respectively) binding to pattern recognition receptors such as Toll-like receptors (TLRs). This interaction triggers the innate immune mechanisms for the rapid clearance of microbes and infected cells, such as phagocytosis, the respiratory burst, the production of proinflammatory cytokines and upregulation of co-stimulatory molecules and MHC-II (25). In contrast, MDSC are the result of prolonged but weak stimulation with growth factors, cytokines and other stimuli (such as GM-CSF, VEGF, NLRP3, and the S100A8/A9 alarmins), and activation is triggered by further stimulation with inflammatory cytokines and pathogen- or damage-associated molecular patterns including iIFN-γ, interleukin (IL)-1β, IL-4, IL-6, IL-13, tumor necrosis factor (TNF), high mobility group box (HMGB)-1 (37–41). The transcription factor STAT3 is also invariably upregulated in MDSC, as the main regulator of genes controlling the expansion of MDSC (31, 42).

Overall, the most important characteristic of these cells which distinguishes them from classical neutrophils and monocytes is their ability to inhibit immune responses, specifically T cell activation and function (28, 31). They use multiple mechanisms to achieve their suppressive effect. Depletion of L-arginine from the microenvironment through upregulation of arginase-1 (ARG1) and inducible nitric oxide synthase (iNOS), inhibits the key human T cell receptor (TCR) signaling molecule CD3ζ *in vitro* (43, 44). Nitric oxide from the iNOS also interferes with JAK/STAT signaling in the T cells of mice *in vitro* (45, 46). In addition to this, increased reactive oxygen species (ROS) in murine immature myeloid cells and human low density granulocytes reduces the expression of CD3ζ on T cells, and reactive nitrogen species (RNS) block T cell activation *ex vivo* by nitrating the TCR and CD8 molecule (47–49). MDSC of both humans and mice express programmed death-ligand-1 (PD-L1) which causes T cell dysfunction, exhaustion and IL-10 secretion when it interacts with programmed death protein-1 (PD-1) on the T cell surface *ex vivo* (50–52). Lastly, in murine models MDSC exert their immunosuppressive effects by the secretion of TGF-β and IL-10, which directly suppress T cells, induce differentiation into T regs, and suppress macrophage IL-12 production. Expression of membrane-bound TGF-β also suppresses NK cells (53–55).

Several host-directed therapies which target MDSC have shown efficacy in cancer therapy, through several mechanisms. MDSC expansion and recruitment can be inhibited with, for example, 5-Fluorouracil or tyrosine kinase inhibitors (56, 57). MDSC function can be inhibited through, for example, phosphodiesterase-5 inhibitors or PD-L1 inhibitors (58, 59). Lastly, agents such as All-trans retinoic acid (ATRA) promote the differentiation of MDSC into mature leukocytes or tumor-specific cells (60). Several of these MDSC targeting agents are in the experimental stages of investigation for use in TB (61, 62). Tasquinimod causes exhaustion of MDSC, and has been shown to enhance mycobacterial clearance in mice (63). Both sildenafil and ATRA initially showed promise as TB host directed therapies, but recent data using human MDSC have been disappointing (64, 65). Many of these MDSC-targeting therapies have been identified as possible treatments for COVID-19, but little data is available on their efficacy (66). The studies in cancer and TB demonstrate the complexity of MDSC. It is likely that particular MDSC subsets predominate in particular conditions, and that they employ different suppressive mechanisms in different disease microenvironments (64). This means that even if an agent is effective in treating one condition, it may not be equally effective in treating a second condition, even if the two conditions have a similar immunopathogenesis. This must be kept in mind for SARS-CoV-2 infection as well.

## 3 MDSC in tuberculosis

Classically activated myeloid cells are the initial effectors of antimycobacterial responses. They sense Mtb through multiple PRRs, phagocytose the bacteria, contain them, limit replication, kill them, release cytokines and chemokines, and activate T cells which in turn increase the activation state of the myeloid cells to enable then to kill Mtb more effectively. However, while alternatively activated myeloid cell subsets – initially labelled natural suppressor cells but later renamed MDSC – also phagocytose Mtb, they have less effective mycobactericidal activity, low expression of MHC class II, secrete immune mediators which suppress T cell responses, and promote lung damage (17, 62, 67, 68).

Mtb contains structural moieties such as glycolipids, that are known to induce MDSC generation (69). MDSC have been detected in the blood of BCG vaccinated mice, where they were found to reduce T cell priming through an IL-1R-dependent pathway (70). Where mice are in the advanced clinical stages of TB disease, MDSC accumulate in the lungs, bone marrow, spleen, and blood, and suppress T cell proliferation and IFN-γ production *in vitro* through NO-dependent mechanisms (71). In humans, MDSC are enriched in the peripheral blood, bronchoalveolar lavage fluid (BALF), and pleural fluid of patients with active pulmonary and pleural TB disease, to levels and phenotype comparable to lung cancer (17, 72). The predominant subset seems to depend on the anatomical site, as PMN-MDSC were preferentially found in BALF, but M-MDSC were the main subset in the pleural fluid (17, 72). Moreover, after successful TB treatment, not only do peripheral blood levels of MDSC decline (particularly PMN-MDSC), but the MDSC express more maturation surface markers (17, 72). During active TB disease, MDSC inhibit T cell proliferation (possibly through a NO-dependent mechanism), suppress CD4^+^ T-cell production of IL-2, TNF-α, IFN-γ *in vitro* (17). MDSC also inhibit IL-10 production by CD4^+^ T cells, thereby inhibiting the regulation of IL-2, TNF and IFNs, and perhaps demonstrating the global shutdown of T cell activation regardless of which cytokines they produce. Furthermore, MDSC suppress CD8^+^ T cell production of TNF-α, IL-2, IFN-γ, and IL-10 in active TB disease, and subvert effector T-cell-mediated containment of Mtb in monocyte-derived macrophages (17, 72, 73). MDSC impair T cell-mediated killing of Mtb infected cells through down-regulation of Th1 cytokines (17, 70). In addition, CD8^+^ T cell mediated killing of infected cells using cytotoxic granules such as perforin and granulysin, is critical for Mtb control (74). Through skewing of the immune response toward a regulatory phenotype, MDSC likely suppress granule-associated effector molecules, and thereby impair killing of infected cells (74–76).

Some of the most compelling evidence for MDSC’s role in TB comes from granuloma research. In mouse models, MDSC accumulate at the edges of necrotic granulomas in the lung parenchyma of infected Mtb-susceptible mice, and this finding has been associated with TB disease progression and uncontrolled bacterial replication (62, 68). Conversely, mice that are Mtb-resistant, with no necrotic granulomas, have very low levels of MDSC in their lungs (68, 77). Suppressive neutrophils which exhibit immunoregulatory functions resembling MDSC subsets have also been identified in TB granulomas from non-human primates (NHPs) (78). Another recent report in NHPs suggested that PMN-MDSC in the periphery of TB granulomas may restrict T cell access to the granuloma core and Mtb-infected cells (79). In an *in vitro* human granuloma model, *ex vivo* generated human M-MDSC promoted mycobacterial replication, changing the structure of the granuloma and adversely affecting bacterial containment (80). In lymph node granulomas from TB/HIV coinfected people and TB-only controls, MDSC expressing Arg-1 were highly expressed in TB/HIV granulomas (81), and the proportion of CD15^+^ MDSC correlated with plasma HIV viral load and Mtb antigen load in tissue, but was negatively correlated with peripheral CD4^+^ T cell numbers. In the same study, PMN-MDSC were also elevated in blood samples from TB/HIV coinfected patients (81). Recently, multiplexed ion beam imaging by time of flight (MIBI-TOF) was used to generate a comprehensive spatial map of 19 cell subsets across 8 spatial microenvironments within TB granulomas from multiple human tissues, including the lung (82). The myeloid core of the granulomas was characterized by expression of the tolerogenic proteins IDO1 and PD-L1, which was highest in CD11b^+^CD11c^+^ macrophages (identical to immunosuppressive TAMs). This expression was also associated with downregulation of HLA-DR in the ‘intermediate monocyte’ subset. These data support the existing evidence for a highly localized, myeloid-mediated immune suppression in the granuloma (82). These findings also seem to support the hypothesis that MDSC which enter a granuloma differentiate into suppressive macrophages which are permissive to Mtb growth, similar to their activity in solid tumors (74, 83).

The role of MDSC in events early in the TB disease spectrum is less clear. Recent attempts to define more clearly the spectrum and pathogenesis of TB before clinical disease highlight the gaps in knowledge of factors promoting progression from infection to disease or to cure (84). Household contacts and people with presumed LTBI have far lower median frequencies of MDSC in peripheral blood than those with active TB disease, comparable to healthy donors (72, 73, 85). Higher frequencies of peripheral blood M-MDSC have been correlated with more severe TB disease (based on time to positivity of Mtb culture, cavitary disease on chest radiography, symptoms score, ESR and monocyte/lymphocyte ratio), but higher frequencies of PMN-MDSC have been associated with a lower radiological TB severity score (85, 86). This suggests that when considering the early TB spectrum, which includes such entities as subclinical TB – where there are no symptoms but may be radiographic changes, and which may or may not progress to active TB disease – the function of the subsets of MDSC need to be examined separately.

## 4 MDSC in COVID-19

As with TB and other viral infections, myeloid cells are the first responders to infection with SARS-CoV-2. After navigating the upper airways, the virus is taken up by alveolar macrophages, without active viral replication, which become activated, and are subsequently responsible for the proinflammatory anti-viral immune response (87). Other innate cells are recruited to the site of infection, and in most people the result is mild disease with eventual eradication of the virus. Yet, a proportion of people infected with SARS-CoV-2 will suffer a marked dysregulation of the innate immune response, especially the myeloid cell compartment, as a result of emergency myelopoiesis (88). This dysregulated state is characterized by the emergence of immature neutrophils and monocytes with suppressive features, including MDSC, which have been directly implicated in the pathogenesis of this dysregulated response, as well as shown to be predictive of severe or fatal disease (88–91).

As we have come to expect from the pandemic literature, there is an abundance of evidence on the topic of MDSC in COVID-19. Several studies in humans have now provided direct evidence of high peripheral blood frequencies of both subsets of MDSC in COVID-19, across all levels of COVID-19 severity but particularly in severe disease, fatal disease, and acute respiratory distress syndrome (ARDS) (91–101). Again, the different roles of the MDSC subsets are evident. Early M-MDSC frequency predicted subsequent COVID-19 severity and mortality, but transient early expansion of PMN-MDSC was associated with survivors of severe COVID-19 (91, 99, 102–104). Studies using single cell RNA sequencing (scRNAseq) in combination with flow cytometry, CyTOF and other assays, have found that populations of immature neutrophils with features of PMN-MDSC and, to a lesser extent, monocytes with features of M-MDSC, emerge in the blood and BALF of patients with severe COVID-19. These cells differentiate them from patients with mild COVID-19, in whom frequencies are still higher than healthy donors (15, 88, 105–107). M-MDSC are not increased in airway samples from nasopharyngeal and endotracheal aspirates, but large numbers of CD66b^+^ cells with a high expression of intracytoplasmic Arg1, in line with PMN-MDSC, were found in lung tissue from patients who died of COVID-19 (91, 97).

The immunosuppressive abilities of these SARS-CoV-2-induced MDSC have been demonstrated. Both PMN-MDSC and M-MDSC from the peripheral blood of patients with moderate and severe COVID-19 inhibit T cell proliferation and IFN-γ production *in vitro* (91, 94–96, 108). In bacterial sepsis, MDSC expansion, IFN-γ production, and TNF-α production reduced over time from admission, but in COVID-19 these responses accelerated over time, despite initial lesser physiological derangement (109). The presence of PMN-MDSC increased the expression of Arg1 and iNOS mRNA compared to PMN-MDSC-depleted PBMC, and plasma levels of TGF-β directly correlated with PMN-MDSC frequency. Moreover, PMN-MDSC depletion significantly improved the SARS-CoV-2 specific T cell response of PBMC (95). Similarly, PD-L1, ILT-3 and IDO-1-expressing M-MDSC were the dominant producers of IL-10 and IL-6 in severe COVID-19 patients, and this correlated with increased inflammatory markers, as well as accumulation of regulatory T and B cells (110). Cocultures with M-MDSC had high levels of Arg1, the suppressive effect of M-MDSC on T cell proliferation was reduced by the addition of L-Arginine, and plasma levels of Arg-1 and IL-6 were elevated in COVID-19 patients, which increased with increasing severity of disease (91). Several other papers have also reported elevated levels of Arg1, with low plasma levels of L-Arginine in association with MDSC in COVID-19, which may not only have implications for immune function but also for increased platelet aggregation (97, 111–113).

Another consideration is the influence of MDSC on the genesis of lung fibrosis in COVID-19 patients. MDSC can transdifferentiate into extracellular matrix (collagen type I)-producing fibrocytes, which interact with activated T-cells, resulting in the production of IDO and leading to Treg expansion (114). Murine models have suggested that MDSC promote lung fibrogenesis by inhibiting collagen degradation through TGF-β production (115). Elevated serum levels of TGF-β correlated with lung fibrosis in a cohort of severe COVID-19 patients (108).This study also reported increased serum levels of TGF-β and of MDSC in COVID-19 patients, as well as a significant correlation between COVID-19 severity and serum TGF-β levels; and showed that isolated M-MDSC from these patients produced higher levels of intracellular TGF-β than non-M-MDSC (108).

These effects do not appear to be short-lived. Elevations in PMN-MDSC persist from hospital admission to convalescence, and longer than three months after acute COVID-19 – across all severities, but at higher levels in those with severe compared to mild disease (94, 116, 117). Elevated levels of circulating M-MDSC were found up to seven months after moderate to severe COVID-19, along with elevated levels of the immune checkpoint marker CD86 (characteristic of ongoing immune activation and chronic inflammation) (118). In another report, MDSC numbers had normalized by three months after acute COVID-19, but the immune dysfunction persisted (119). When monocytes from COVID-19 patients at three months after hospital discharge were stimulated with LPS and R848, both TNF and IL-6 production was impaired, and levels of other cytokines in plasma were also lower, in both moderate and severe COVID-19 (119). At five months after SARS-CoV-2 infection, levels of M-MDSC remained elevated compared to healthy controls, and continued to suppress SARS-CoV-2-specific T cell cytokine production through arginase, ROS and TGF-β dependent pathways (120). These data suggest a long-lasting impairment in the immune response after COVID-19, attributable to the suppressive effects of MDSC.

The evidence for MDSC involvement in COVID-19 and TB are summarized in **Table 1**.

**Table 1:** Evidence for the role of myeloid derived suppressor cells in the pathogenesis of Tuberculosis compared to COVID-19.

MDSC in Mtb infection and disease	MDSC in SARS-CoV-2 infection and disease
**Peripheral blood** *In mice* • Raised levels of MDSC in blood of BCG vaccinated mice, and Mtb susceptible mice with advanced TB disease *In humans* • Raised levels of MDSC in blood of humans with active TB disease • High frequencies of M-MDSC correlate with more severe clinical and radiological active TB disease • High frequencies of PMN-MDSC associated with lower radiological severity • Levels decline after successful TB treatment, cells express more maturation markers • Levels in LTBI comparable to healthy donors	**Peripheral blood** • High frequencies of both M-MDSC and PMN-MDSC, highest in severe and fatal COVID-19 • Early M-MDSC levels predict COVID-19 severity and mortality • Transient early expansion of PMN-MDSC associated with surviving severe COVID-19 • High frequencies persist up to 7 months after acute COVID-19 • Convalescent MDSC levels are higher in those recovering from severe COVID-19 than mild COVID-19
**Lungs** *In mice* • Accumulate in lungs (and bone marrow, spleen) in Mtb susceptible mice with active TB disease *In humans* • Accumulate in BALF and pleural fluid of humans with active TB disease • PMN-MDSC dominant in BALF of humans with pulmonary TB • M-MDSC dominant in the pleural fluid of humans with pleural TB disease **Granulomas** *In mice* • Accumulate at the edge of necrotic granulomas • Associated with TB progression and uncontrolled Mtb replication *In humans* • Found in the myeloid core • Promote mycobacterial replication • Impair Mtb containment	**Lungs** • Not increased in nasopharyngeal and endotracheal aspirates • Found in BALF of patients with COVID-19, higher in severe disease • Likely PMN-MDSC found in lung tissue from deceased COVID-19 patients • TGF-β levels correlates with lung fibrosis and COVID-19 severity
**Immunosuppressive mechanisms** *In mice* • Reduce T cell priming through an IL-1R-dependent pathway • Inhibit T cell proliferation and IFN-γ production *in vitro* through NO-dependent mechanisms *In humans* • Upregulated ARG1 and PD-L1 expression • Inhibit T cell proliferation (possibly through a NO-dependent mechanism) • Suppress CD4^+^ T-cell > CD8^+^ T cell production of IL-2, TNF-α, IFN-γ • Suppress CD4^+^ T cell > CD8^+^ T cell production of IL-10 • Subvert effector T-cell-mediated containment of Mtb in monocyte-derived macrophages	**Immunosuppressive mechanisms** • M-MDSC and PMN-MDSC inhibit T cell proliferation and IFN-γ production • Associated with increased expression of ARG1 and iNOS mRNA • Levels of PMN-MDSC correlate with plasma levels of TGF-β • M-MDSC are dominant producers of IL-10 and IL-6 in severe COVID-19 • M-MDSC produce higher levels of TGF-β than other MDSC • M-MDSC correlated with increased inflammatory markers, B cell and Treg accumulation • Associated with high levels of ARG1, low plasma L-Arginine • Addition of L-Arginine partially inhibits suppressive effect of M-MDSC on T cell proliferation • Suppress T cell cytokine production at 5 months after recovery from COVID-19 through ARG1, ROS, and TGF-β dependent pathways

ARG1, arginase-1; BALF, bronchoalveolar lavage fluid; BCG, Bacille Calmette Guerin intradermal vaccine; IFN, interferon; IL-1R, interleukin 1 receptor; iNOS, inducible nitric oxide synthase; LTBI, latent tuberculosis infection; mRNA, messenger ribonucleic acid; Mtb, Mycobacterium tuberculosis; MDSC, myeloid-derived suppressor cells; M-MDSC, monocytic MDSC; NO, nitric oxide; PMN-MDSC, polymorphonuclear MDSC; ROS, reactive oxygen species; TB, tuberculosis; TGF, transforming growth factor; TNF, tumor necrosis factor; Treg, regulatory T cell.

## 5 MDSC in SARS-CoV-2 and Mtb coinfection

**Figure 2:**
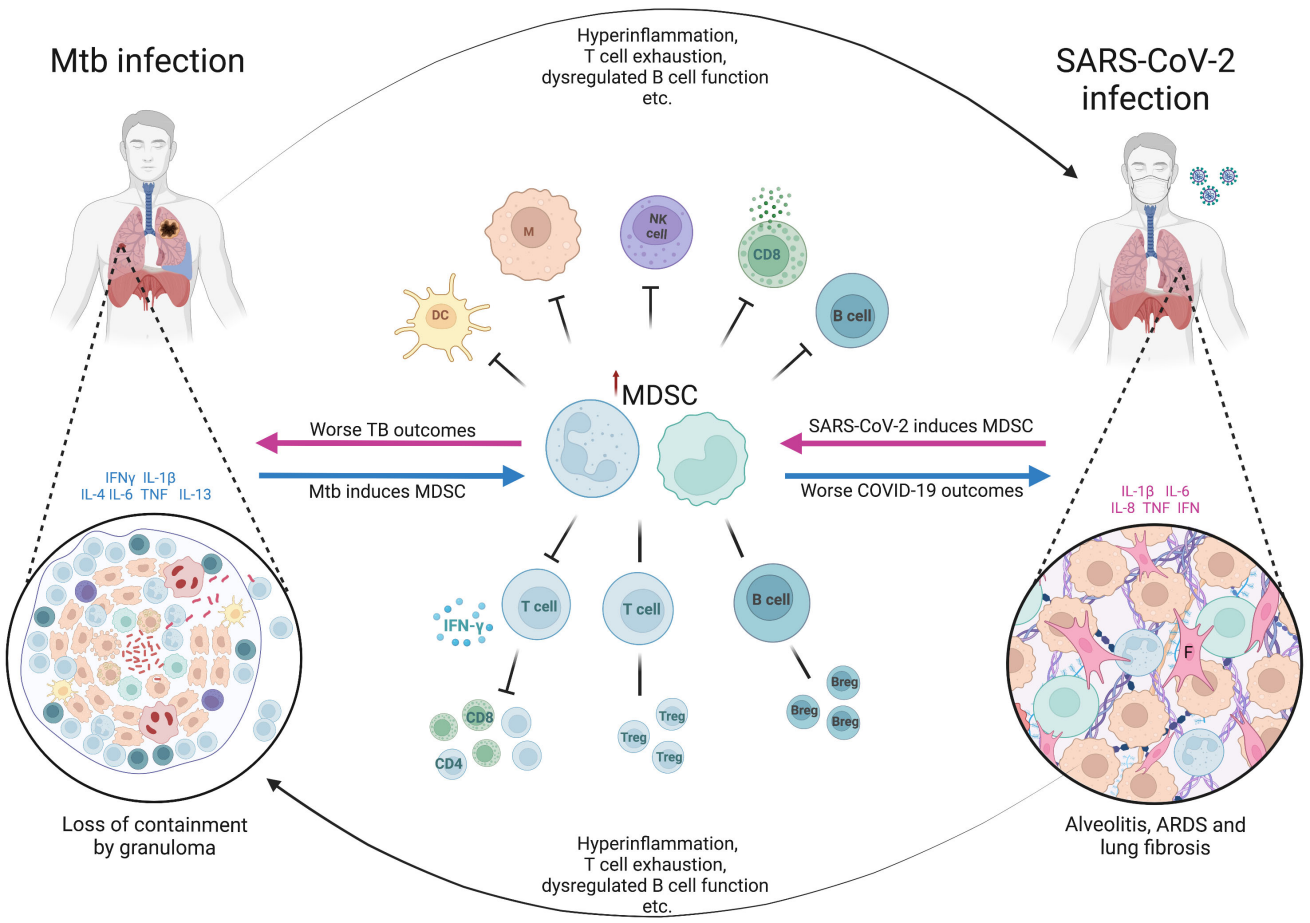
The hypothetical role of myeloid derived suppressor cells in Mtb/SARS-CoV-2 coinfection. The figure shows the bidirectional effects of myeloid derived suppressor cells (MDSC) on the clinical outcomes in patients coinfected with *Mycobacterium tuberculosis* (Mtb) and SARS-CoV-2. In latent infection with Mtb or active TB disease, the presence of cytokines such as IFN-γ, IL-1β, IL-4, IL-6, TNF and IL-13, triggers the pathological activation of MDSC (blue arrows), which become immune suppressive. The effects of MDSC, along with other effects of Mtb infection and disease (hyperinflammation, T cell exhaustion etc.), predispose to worse clinical outcomes from SARS-CoV-2 infection including acute respiratory distress syndrome (ARDS) and lung fibrosis. MDSC accumulate in the lungs of severe SARS-CoV-2 cases (shown in the cut-out on the right), further exacerbating the immune suppression and profibrotic effects. Similarly, in current or recent SARS-CoV-2 infection, there is induction of proinflammatory cytokines, often pathologically high (termed hyperinflammation), along with other aspects of SARS-CoV-2-related immune dysfunction and dysregulation, and pathological activation of MDSC (pink arrows). These factors combined result in worse clinical outcomes from active tuberculosis disease, as well as the potential loss of containment of Mtb within granulomas in latent infection. Suppressive neutrophils and macrophages within the myeloid core of the granuloma (shown in the cut-out on the left) further contribute to the loss of Mtb control. Breg, regulatory B cell; COVID-19, coronavirus disease-2019; DC, dendritic cell; F, fibroblast; IFN, interferon; IL, interleukin; M, macrophage; NK, natural killer cell; TB, tuberculosis; Treg, regulatory T cell. Created with BioRender.com

It is thought that up to a quarter of the world’s population have been latently infected with Mtb, with at most 15% progressing to active disease in their lifetime, often with a long latency period between initial infection and active TB disease (121). This means that a coinfection sequence of pre-existing LTBI followed by SARS-CoV-2 infection is a highly likely scenario. However, the fact that many patients present with a long history of symptoms and cavitary lung lesions at TB diagnosis, implying chronicity of disease, suggests that a scenario of active TB disease followed by SARS-CoV-2 infection is also likely to be a common occurrence (**Figure 2**).

The number of Mtb-specific CD4 T cells is lower in coinfected patients who have active TB disease/SARS-CoV-2, than in those with TB alone (12). Also, COVID-19 itself is characterized by reduced T cell numbers and T cell exhaustion (12, 122). Therefore, SARS-CoV-2 infection could hypothetically trigger progression from LTBI to active TB disease. As we have shown in the sections above, MDSC may be responsible for the lower T cell numbers in COVID-19, and for reduced expression of key cytokines required to maintain the immune response to Mtb.

The levels of IFN-γ produced by whole blood on stimulation by the SARS-CoV-2 spike protein-derived peptide CD4S were lower in patients with active TB disease/COVID-19 coinfection than in those with LTBI/COVID-19 and COVID-19 alone (13). In a more comprehensive analysis by the same group, an immune signature consisting of TNF-α, macrophage inflammatory protein-1β and IL-9 associated with active TB disease/COVID-19 compared to COVID-19 alone, and another signature including TNF-α, IL-1β, IL-17A, IL-5, fibroblast growth factor-basic, and GM-CSF, associated with active TB disease/COVID-19 compared to TB alone (14). In addition to this they confirmed the reduced SARS-CoV-2 specific response in coinfected patients for IFN-γ, as well as IL-10, IP-10, and other key cytokines (14). This suggests the possibility that the pre-existing immune milieu of active TB disease impaired the critical T cell response to SARS-CoV-2 infection. Hypothetically, MDSC induced by Mtb infection may be responsible for this, through the expression of PD-L1 which directly inhibits further T cell proliferation and induces both dysregulation and an exhausted immune profile in T cells. If this is the case, then the clinical implication may be a higher viral load, more severe virus-related lung damage, and an increased immune dysregulation which results in more severe clinical manifestations.

The impact of MDSC on initial or recent SARS-CoV-2 infection with secondary Mtb infection must also be considered. As described in detail above, on infection with SARS-CoV-2, emergency myelopoiesis is stimulated and MDSC released into the peripheral blood, accumulating in the lungs in severe COVID-19. Those who die from COVID-19 will most likely have early and persistently elevated M-MDSC, whereas survivors are more likely to have an early peak of PMN-MDSC which reduces as they improve. Nonetheless, the immune suppression mediated by these cells persists for many months irrespective of severity. In theory, Mtb infection during the acute phase of SARS-CoV-2 illness or in the months thereafter, may therefore have a higher likelihood of a poor outcome, as the key T cell responses to Mtb are impaired. This is supported by reports of deficient IFN-γ release assay (IGRA) responses to Mtb antigens (and mitogen) in patients with severe COVID-19, which suggest a generalized unresponsiveness of T cells to all antigens, and specifically to Mtb (123, 124). Production of IFN-γ, IP-10, and IL-1β, amongst others, in response to Mtb-antigen stimulation was impaired in the whole blood from coinfected active TB disease/COVID-19 patients (14). On the other hand, some data implies that the Mtb-antigen cytokine responses are augmented by recent SARS-CoV-2 infection, and immunosuppressive responses are reduced (125). The latter study examined the baseline, Mtb antigen-, and mitogen-stimulated levels of key cytokines and chemokines in elderly patients with positive and negative SARS-CoV-2 serology, both with and without LTBI (based on IGRA). They found higher baseline/unstimulated and Mtb antigen-stimulated levels of IFN-γ, IL-2, TNF-α, and others, in patients with LTBI who were seropositive for SARS-CoV-2 compared to those with LTBI who were seronegative for SARS-CoV-2. Moreover, the levels of immunosuppressive IL-10 were lower in LTBI/seropositive SARS-CoV-2 individuals. There were no differences in response to mitogen between groups in this study, and the LTBI negative control group did not show any enhanced cytokine response to Mtb antigen (125). These data show that if SARS-CoV-2-induced MDSC are the mechanism underlying the poor response of T cells to Mtb antigen, then their effects are likely Mtb antigen-specific, rather than part of a non-antigen-specific response. These observations also suggest that SARS-CoV-2-induced MDSC may have a different effect on the outcome of Mtb infection, in different COVID-19 severities.

Whilst it is not direct evidence for the effect of MDSC, the fact that Mtb-specific T cell numbers are reduced in SARS-CoV-2 coinfection suggests that if MDSC are involved, then it is in an antigen-specific manner (12). In other words, MDSC induced by SARS-CoV-2 may well suppress Mtb-specific T cell responses, as well as suppressing global T cell proliferation and cytokine production in an antigen non-specific way. Similarly, evidence that there is a reduced T cell response to stimulation with SARS-CoV-2 antigen in patients with preexisting Mtb infection might imply that Mtb-induced MDSC also suppress SARS-CoV-2 T cells in both an antigen specific and non-specific way (14). This is not unknown, as MDSC induced by HIV infection also suppress T cell function *in vitro* by both antigen-specific and non-specific mechanisms (126). However, this antigen-specific effect may be limited to CD8 ^+^ T cells because of the low expression of MHC Class II by MDSC – a theory supported by the inhibitory effects of tumor-associated MDSC on CD8^+^ T cells demonstrated in human cells *in vitro* and mouse models, which can be reversed by anti-MDSC host directed therapies (127, 128).

Whether any of the MDSC-targeting host directed therapies with efficacy in TB will also prove effective in Mtb/SARS-CoV-2 co-infection remains to be seen. Because of the high prevalence of both infections, it is likely that by default coinfected participants will be included in any human trials in the future. An agent such as Imatinib (a tyrosine kinase inhibitor which targets the ABL kinase domain) which has experimental evidence in both diseases – despite disappointing clinical disease outcomes in COVID-19 – is an attractive option for future investigation in coinfection (129–132).

Lastly, we must consider if preexisting Mtb infection (LTBI or active TB disease) with MDSC induction might affect the efficacy of a subsequent SARS-CoV-2 vaccination, and if recent COVID-19 might adversely affect a subsequent Mtb vaccine response. MDSC impair both T and B cell responses, even inducing regulatory and suppressive B cells in the tumor microenvironment (133). Theoretically, the presence of MDSC from Mtb or SARS-CoV-2 infection might impair adaptive and cell-mediated responses to a vaccine administered in their presence, resulting in reduced immune memory to the vaccine (133). Adults with TB have lower levels of anti-SARS-CoV-2 antibodies after three doses of inactivated vaccine (134). Assuming antibody levels are a correlate of protection, this would mean that a SARS-CoV-2 vaccinated person with preexisting Mtb infection would lose the vaccine-mediated protection for severe COVID-19, and lead to worse clinical outcomes. MDSC are known to be part of the response to Bacillus Calmette-Guérin vaccination (BCG), possibly contributing to incomplete protection against Mtb by restraining T cell priming (70). BCG has immunomodulatory effects on myeloid cells, including epigenetic reprogramming, which affect these cells’ ability to respond to pathogens (including Mtb) (135, 136). A person receiving BCG or another Mtb vaccine candidate in a clinical trial may not develop the desired immune response if they have had recent COVID-19, and importantly, the SARS-CoV-2-induced MDSCs continue to exert their effects months after the acute infection. This would need to be adjusted for in the analysis.

Overall, there is evidence supporting a potential role for MDSC in determining the outcomes of Mtb/SARS-CoV-2 coinfection, in all disease severities and iterations of the coinfection sequence. More evidence is needed to find out if MDSC impact the outcome of SARS-CoV-2 infection in early TB or LTBI; if there are any vaccine-relevant interactions; and if a host directed therapy aimed at modulating the effect of MDSC might improve the outcome of active TB disease/COVID-19 coinfection.

## Data availability statement

The original contributions presented in the study are included in the article/supplementary material. Further inquiries can be directed to the corresponding author.

## Author contributions

NdP, GW, SM and JS conceived of the article, JS wrote the first draft of the manuscript and all authors edited and approved the final version.
